# Electron tunneling through double magnetic barriers in Weyl semimetals

**DOI:** 10.1038/s41598-017-12835-0

**Published:** 2017-10-19

**Authors:** Xunwu Hu, Fang Cheng

**Affiliations:** Department of Physics and Electronic Science, Changsha University of Science and Technology, Changsha, 410004 China

## Abstract

We theoretically investigate the transport in a magnetic/normal/magetic hybrid structure on the surface of a Weyl semimetal. We find a directional-dependent tunneling which is sensitive to the magnetic field configuration and the electric gate voltage. The momentum filtering behavior becomes more significant for two-delta-function-shaped magnetic barriers. There are many Fabry-Pérot resonances in the transmission determined by the distance between the two magnetic barriers. The combined effects of the magnetic field and the electrostatic potential can enhance the difference in the transmission between the parallel and antiparallel magnetization configurations, and consequently lead to a giant magnetoresistance.

## Introduction

Topological Weyl semimetals have sparked tremendous recent interest in condensed matter physics^1,2^. These materials host low energy excitations with massless, linear dispersions around nodes, termed the Weyl points, as the three-dimensional (3D) analogue of graphene. One of the most distinctive features of the system is the coexistence of the topological surface states and the bulk massless Fermion states. 3D Dirac semimetals have been realized in Na_3_Bi^3,4^, Cd_3_As_2_^5–9^ and ZrTe_5_^10,11^. The recent theoretical predictions^12–14^ and experimental discoveries^15–17^ of Weyl semimetals open up an exciting new solid state playground for exploring the physics of anomalous quantum field theories. Moreover, due to the high mobility and chiral nature of electrons in Weyl semimetals, they are expected to be ideal candidates for transport and tunneling applications. Several transport applications such as charge transport^18,19^, magnetotransport^20,21^, extremely large magnetoresistance and ultrahigh mobility^22^ have been predicted and observed recently.

In this work, we study electron tunneling through two types of magnetic double barriers where we considered square-shaped and delta-function-shaped magnetic fields. The square-shaped magnetic fields can be created by depositing superconducting strips above the Weyl semimetal in the presence of a magnetic field, neglecting the shrinking effect at the edges^23^. The upper limit of the magnetic barrier strength induced by a uniform magnetic field on superconductor pattern is restricted by the critical magnetization of the superconductor material, which is in excess of 30 T, as shown in Nb3Sn^24^. The delta-function-shaped magnetic field is a simplified model for the magnetic field profile created by depositing ferromagnetic metallic strips on the surface of a Weyl semimetal with the magnetization parallel to the surface. The ferromagnetic strips are electrically isolated from the Weyl semimetal through, e.g., a thin oxide layer^25,26^. The upper limit of the magnetic field strength induced by ferromagnetic stripes directly depends on the saturation magnetization of the ferromagnetic material. For instance, the saturation magnetization of magnetic barrier around 3.75 T has been experimentally achieved, and the transport properties under the influence of the resulting magnetic barrier have been investigated in two-dimensional electron gas structures^27^. It is experimentally shown that in 3D Dirac semimetals, the Fermi level as well as potential barrier height, can be tuned by applying a bias to a gated region or by alkali metal doping^28,29^.

We investigate theoretically the transmission and conductance for parallel and antiparallel magnetic field configurations. We find that the transmission of electrons through the double barrier structures depends sensitively on the incident angles, the Fermi energy, the magnetic fields, and the electric gate voltages. The tunneling processes exhibit momentum filtering behavior caused by the inhomogeneous magnetic field. The tunneling magnetoresistance in such systems can be tuned significantly by changing the magnetic field and the height of the electric potential.

The paper is organized as follows. In Sec. II we present the theory of electron tunneling through magnetic and electric double barrier structures. In Sec. III, we show the numerical results and present our discussions. Finally, we give a brief conclusion in Sec. IV.

## Methods

**Figure 1:**
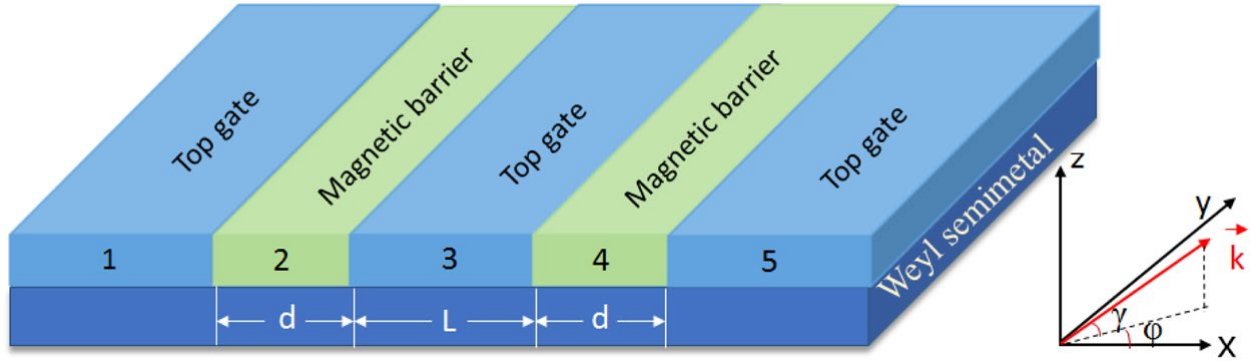
The schematic representation of the double magnetic barrier structure on the top surface of a Weyl semimetal. The red arrow represents incident wavevector of the Weyl Fermions with angle *γ* and *φ*.

We consider a double magnetic barrier with square-shaped or delta-function-shaped magnetic field on the surface of a Weyl semimetal, as shown in Fig. 1. The square-shaped magnetic fields can be created by depositing superconducting strips above the Weyl semimetal in the presence of a magnetic field, neglecting the shrinking effect at the edges. While the delta-function-shaped magnetic field is a simplified model for the magnetic field profile created by depositing ferromagnetic metallic strips on the surface of a Weyl semimetal with the magnetization parallel to the surface. In all cases, the created relevant magnetic fields are directed perpendicular to the surface of the Weyl semimetal. The low energy Dirac Hamiltonian of the Weyl fermion can be described as follows

1 $$H={v}_{F}\,(\sigma \cdot ({\bf{p}}+e{\bf{A}}{\boldsymbol{)}})+V,$$

where *v*_*F*_ is the Fermi velocity, and *σ* represents Pauli matrices, *V* is the height of the electrostatic barrier. **B**(*x*) = (0, 0, *B*). **A** = (0, *A*_*y*_, 0) is the vector potential generated by the magnetic metal strips. To investigate the transport properties in the bulk system, we consider Weyl electrons near one node and neglect the contribution of surface states (and hence Fermi arcs) and intervalley scattering to the conduction. Note that the Zeeman term affects the transmission slightly at low magnetic field. Here, we have neglected the Zeeman splitting since the band shift is very small and only observable under a very high magnetic field of ≈25 T, as shown experimentally in the Weyl semimetal TaP^30^. The Hamiltonian shown in Eq. 1 describes the dynamics of low energy electrons near one node in Weyl semimetals. Therefore the validity of this approximation is restricted by an energy range depends on the full band structure of the material hosting such a Weyl nodes with linear energy dispersion. For simplicity, we introduce dimensionless units: *l*_*B*_ = [*ħ*/*eB*_0_]^1/2^, *E*_0_ = *ħv*_*F*_/*l*_*B*_, *r* → *l*_*B*_ *r*, *k* → *k*/*l*_*B*_, *B*(*x*) → *B*_0_ *B*(*x*), *E* → *E*_0_ *E*, the Hamiltonian becomes

2 $$H=(\begin{array}{cc}V+{k}_{z} & {k}_{x}-i\,({k}_{y}+{A}_{y})\\ {k}_{x}+i\,({k}_{y}+{A}_{y}) & V-{k}_{z}\end{array}),$$

where the three components of wavevector can be written as *k*_*x*_ = *k*_*F*_ cos *γ* cos *φ*, *k*_*y*_ = *k*_*F*_ cos *γ* sin *φ*, *k*_*z*_ = *k*_*F*_ sin *γ* with the Fermi wavevector ${k}_{F}={k}_{x}^{2}+{k}_{y}^{2}+{k}_{z}^{2}$. By solving the above Hamiltonian, the components of the wave vector satisfy the relationship: ${(E-V)}^{2}={k}_{x}^{2}+{({k}_{y}+{A}_{y})}^{2}+{k}_{z}^{2}$.

In order to understand the effect of the magnetic field configurations on the electron tunneling, we consider two different magnetic field profiles, i.e., square-shaped and delta-function-shaped magnetic fields. For a square-shaped magnetic barrier, the magnetic field is *B*_*z*_(*x*) = *B*[(*θ*(*x* − *d*) − *θ*(*x*))/2 + *γ*(*θ*(*x* − 2*d* − *L*) − *θ*(*x* − *d* − *L*))/2] with *γ* = ±1 representing the magnetization configuration, and the vector potential is a linear function in the barrier regions. To construct the wave function in each region we utilize the symmetries of the system. The momentum *p*_*y*_ and *p*_*z*_ along the interface are good quantum numbers because of the translational invariance along the *y* and *z* direction. Therefore the wave function can be separated ${\rm{\Phi }}(x,y)={\rm{\Psi }}(x){e}^{i{k}_{y}y+i{k}_{z}z}$, where *k*_*y*_ and *k*_*z*_ are the wave numbers along the *y* and *z* direction, respectively. In the free region 1, the vector potential is a constant, the corresponding energy *E*_±_ = ±*k*_*F*_, and the wave function is

3 $${{\rm{\Psi }}}_{1}=\frac{1}{\sqrt{2}}\,(\begin{array}{c}1\\ \tfrac{{k}_{x}+i{k}_{y}}{E+{k}_{z}}\end{array})\,{e}^{i{k}_{x}x}+\frac{r}{\sqrt{2}}\,(\begin{array}{c}1\\ \tfrac{-{k}_{x}+i{k}_{y}}{E+{k}_{z}}\end{array})\,{e}^{-i{k}_{x}x}.$$

In the barrier region 2, the vector potential is given by *A* = (0, *Bx*, 0) and the wave function can be expressed in terms of the parabolic cylinder functions *D*_*v*_,

4 $${{\rm{\Psi }}}_{2}=\sum _{\pm }\,{c}_{\pm }\,(\begin{array}{c}{D}_{v\mathrm{/2}-1}\,(\sqrt{2}\,({k}_{y}+{A}_{y}))\\ \pm i\sqrt{\mathrm{2/}v}{D}_{v\mathrm{/2}}\,(\sqrt{2}\,({k}_{y}+{A}_{y}))\end{array}),$$

with the complex coefficients *c*_±_ and $v={({E}_{F}-V)}^{2}-{k}_{z}^{2}$. In regions 3, 4, and 5, the corresponding wave functions can be obtained by repeating a similar procedure. For a delta-shaped magnetic barrier, the magnetic field is perpendicular to the surface and given by *B*_*z*_(*x*) = *B*[(*δ*(*x*) − *δ*(*x* − *d*))/2 + *γ*(*δ*(*x* − *d* − *L*) − *δ*(*x* − 2*d* − *L*))/2] and the vector potential is constant in the barrier regions. The electron wave function for the delta-shaped magnetic barrier is similar to that of the square-shaped magnetic barriers obtained above, but with the different vector potential A_*y*_. We have shown the values of the vector potential A_*y*_ in the different regions for the different magnetic barrier configurations in Table 1. Using the scattering-matrix technique, we obtain the transmission probability. In this work, we adopt the magnetic unit *B*_0_ = 1*T*, the energy unit *E*_0_ = 16 *meV*, and the length unit *l*_*B*_ = 26 *nm*.

**Table 1:** The magnitude of the vector potential A_*y*_ in different magnetic barrier configurations.

Structure		A*y*				
		Region 1	Region 2	Region 3	Region 4	Region 5
Square-shaped magnetic barriers	parallel configuration	0	Bx	Bd	B(x − L)	2Bd
	antiparallel configuration	0	Bx	Bd	B(2d + L − x)	0
Delta-shaped magnetic barriers	parallel configuration	0	Bd	0	Bd	0
	antiparallel configuration	0	Bd	0	−Bd	0

## Results and Discussions

### Transmission through double square-shaped magnetic barriers

**Figure 2:**
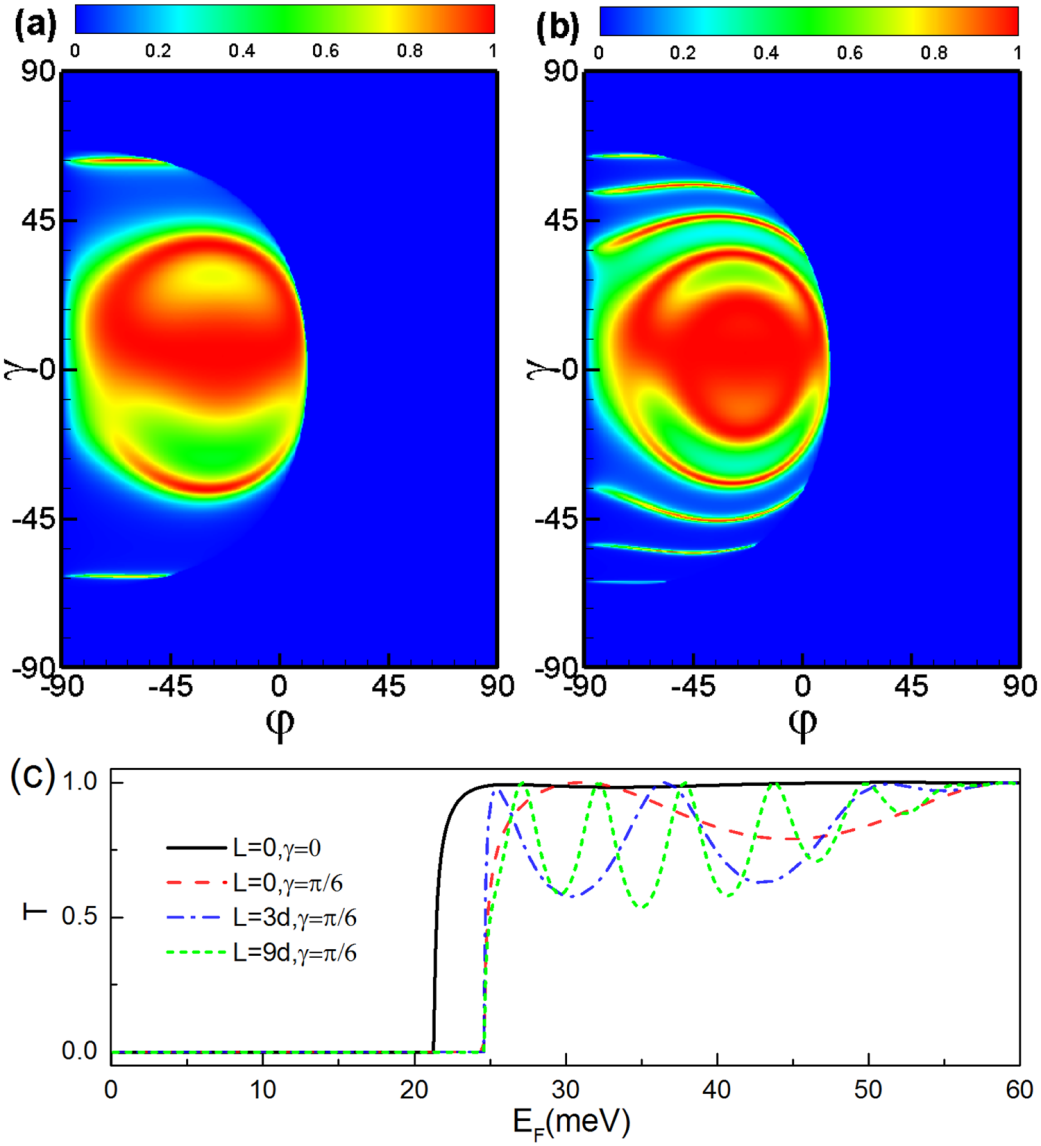
(**a**) The contour plot of the transmission probability through a parallel square-shaped magnetic double barrier as a function of the incident angle *φ* and *γ*, for incident energy *E*_*F*_ = 40 meV and a fixed barrier width *d* = 26 nm, distance *L* = 3*d*, and magnetic field *B* = 1 T. (**b**) The same as panel (a) but for the distance *L* = 9*d*. (**c**) Transmission probability as a function of the incident energy *E*_*F*_ for a fixed incident angle *φ* = −*π*/6, different incident angle *γ* and distances between the two magnetic barriers.

First we consider the tunneling process through double square-shaped magnetic barriers with parallel configuration. Figure 2 shows angular (*φ*, *γ*) dependence of transmission probability in the case of *E*_*F*_ = 40 meV, a magnetic field *B* = 1 T, the width *d* = 26 nm. The perfect transmission rings are deflected and distorted in the presence of the magnetic field, as shown in Fig. 2(a,b). From the contour plot of the transmission as function of the incident angles *φ* and *γ*, one can see clearly the transmitted window of electron tunneling through the magnetic barriers. We find that the transmission become asymmetric with respect to the incident angles *φ* and *γ*, which is induced by the inhomogeneous magnetic field. It is interesting to notice that tunneling is forbidden for certain incident angles (*φ*, *γ*), i.e., a wave-vector filtering behavior is found. The total reflection for the parallel configuration stems from the evanescent modes in the outgoing region 5. The boundary of the total reflection region *T* = 0 can be approximately given by the relationship *E*_*F*_ cos *γ*(1 − sin *φ*) = 2*Bd*. For a fixed incident energy, the transmission declines sharply and vanishes when the incident angle *φ* (*γ*) exceeds a critical value *φ*_*c*_ = arcsin (1 − *A*_*y*_/(*E*_*F*_ cos *γ*)) (*γ*_*c*_ = arccos (*A*_*y*_/(*E*_*F*_(1 – sin *φ*))). This is because the wavevector in the right-side of the barrier become imaginary denoting the appearance of evanescent modes. Figure 2(c) shows the transmission as a function of different distances *L*. For a single barrier structure (i.e., *L* = 0) with *γ* = 0, there is no oscillating behavior because no quasibound states exist between the two barriers. However, here we found oscillating behavior for *L* = 0 and *γ* = *π*/6, because there is quasibound states between the two barriers induced by the reflection in the *z* direction. When *L* ≠ 0 and *γ* ≠ 0, there is Fabry-Pérot modes formed between the two barriers due to the multiple reflections between the two interfaces in the *y* and *z* directions, thus the oscillation becomes more pronounced as the distance between the two barriers *L* increases.

**Figure 3:**
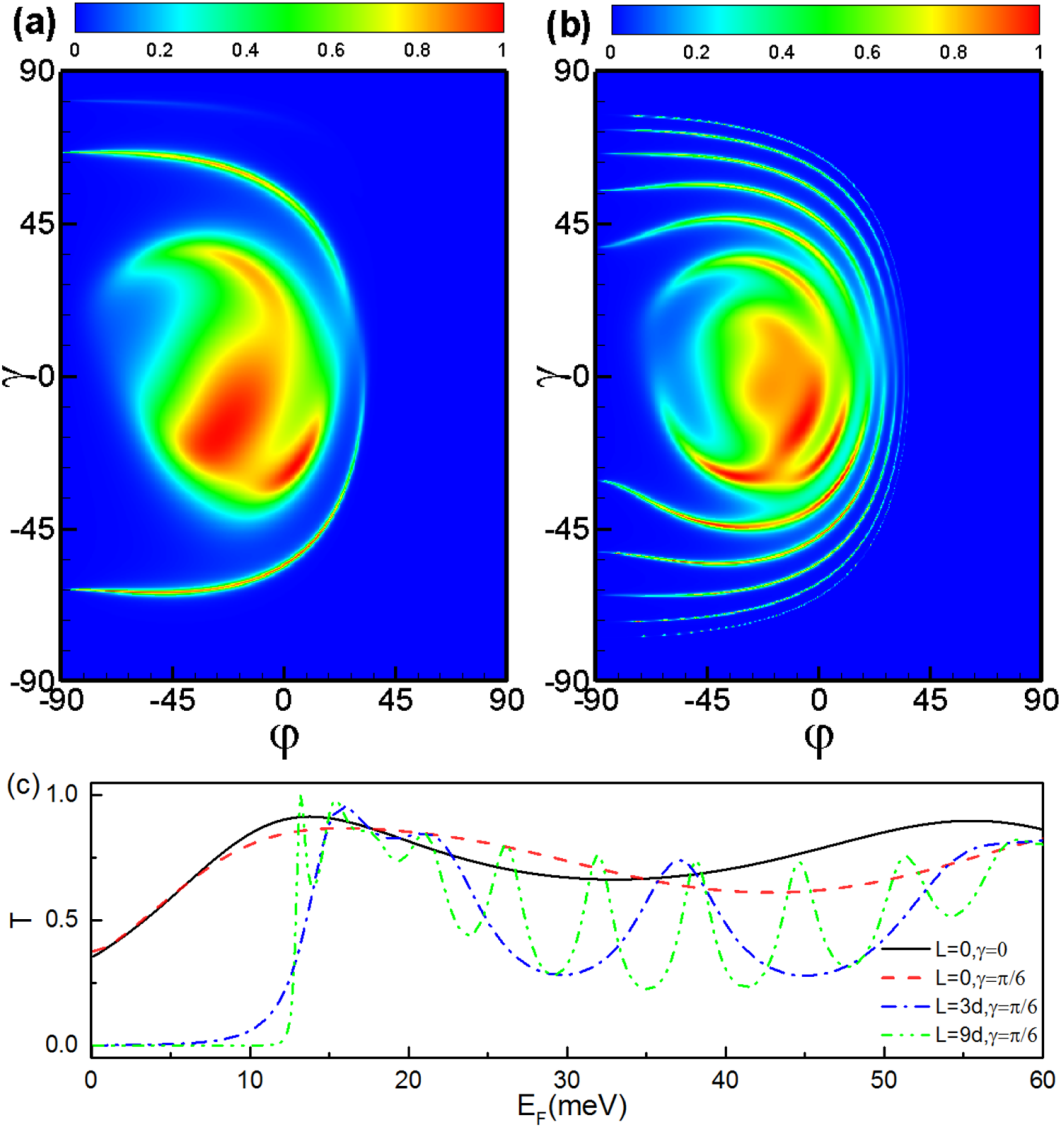
The same as Fig. 2 but for the antiparallel configuration.

Next we discuss the transmission through double square-shaped magnetic barriers with antiparallel configuration. The transmission (see Fig. 3(a,b)) becomes very different from that of the parallel configuration. The total reflection for the antiparallel configuration stems from the evanescent modes in the middle region 3. The boundary of the tunneling forbidden region can be approximately given by the relationship *E*_*F*_ cos *γ*(1 − sin *φ*) = *Bd*. Electrons can tunnel through the double barrier structure with the antiparallel configuration even when the electron wave vector *k*_*x*_ is imaginary, i.e., evanescent modes, in the middle region between the two barriers. It is in that the vector potentials are the same on each side of the double barrier, i.e., the antiparallel configuration, the outgoing wave is always in the propagating modes. Figure 3(c) shows the transmission as a function of the incident energy at different distances between two barriers. Notice that there are more pronounced resonant peaks caused by the Fabry-Pérot modes formed between the two barriers with increasing distance *L* than that for the parallel configuration case.

**Figure 4:**
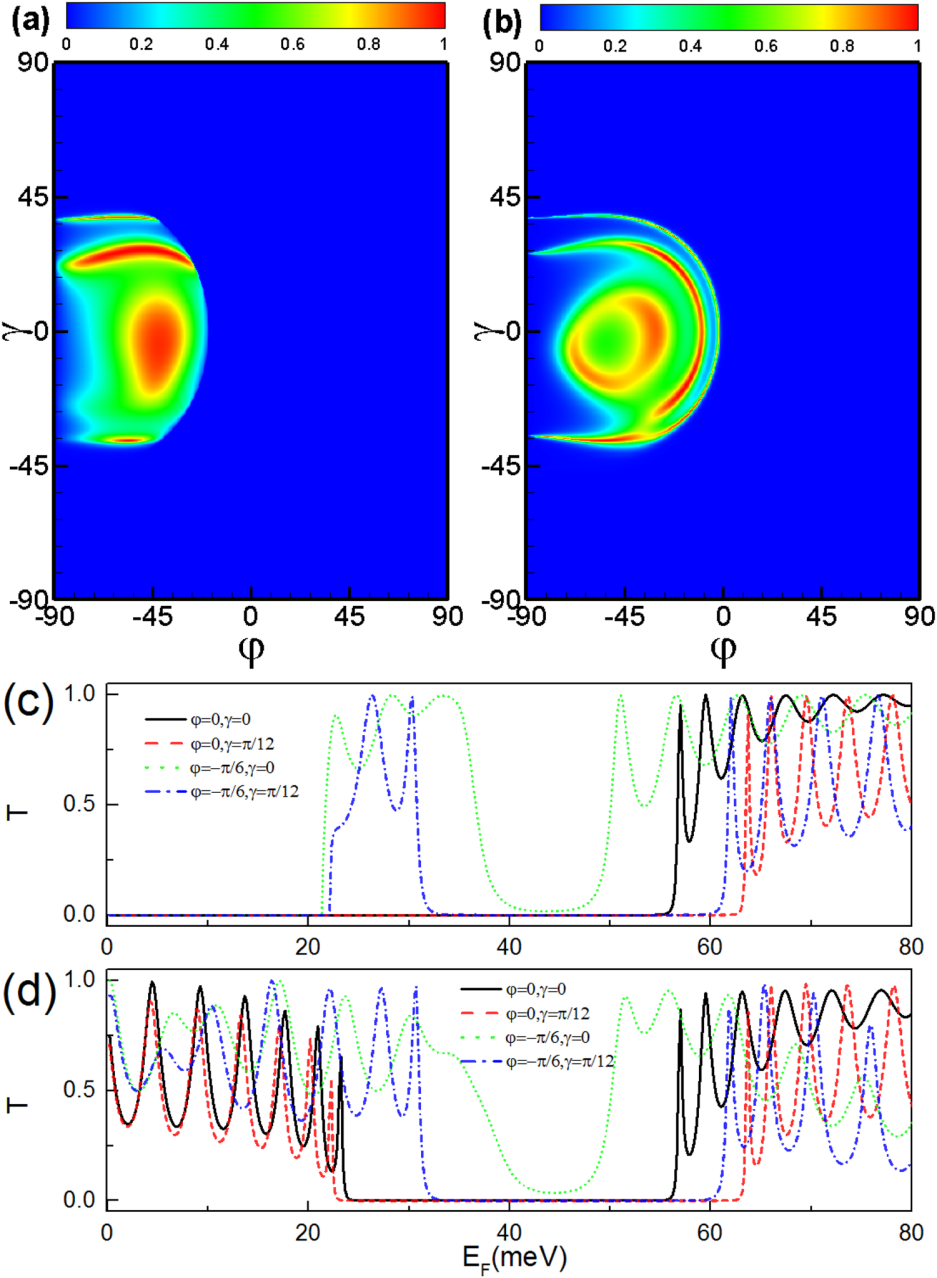
Transmission probability through a parallel square-shaped magnetic double barrier as a function of the incident angle *φ* and *γ*, for incident energy *V*_3_ = 40 meV, incident energy *E*_*F*_ = 24 meV. (**b**) The same as panel (a) but for the antiparallel configuration. (**c**) Transmission probability as a function of the incident energy *E*_*F*_ for different incident angle with a potential barrier *V*_3_ = 40 meV. (**d**) The same as panel (a) but for the antiparallel configuration. The distance is L = 9d = 234 nm.

It is interesting to see the effect of the electrostatic potential on the perfect transmission and total reflection. In Fig. 4, we plot the transmission probability as a function of the incident angles (*φ*, *γ*) and incident energy *E*_*F*_ in case of *V*_3_ = 40 meV applied in region 3. Figure 4(a,c) for double square-shaped magnetic barriers with parallel configuration, while Fig. 4(b,d) for double square-shaped magnetic barriers with antiparallel configuration, respectively. It is interesting to notice that for a fixed *φ*, the tunneling is forbidden for a wider region when the incident angle *γ* increases. In contrast, for a fixed *γ*, the tunneling is forbidden for a narrower region when the incident angle *φ* increases. (see Fig. 4(c,d)) According to the relation ${k}_{x}=\sqrt{{({E}_{F}-V)}^{2}\,{\cos }^{2}\,\gamma -{({E}_{F}\cos \gamma \sin \phi +{A}_{y})}^{2}}$, the wave vector *k*_*x*_ tends to be imaginary as the incident angle *γ* increases or *φ* decreases. The total reflection for the parallel and antiparallel configuration in the presence of *V*_3_ stems from the evanescent modes in the middle region 3 and outgoing region 5. When *φ* ≠ 0 and *γ* ≠ 0, the tunneling is forbidden for the energy satisfying ${({E}_{F}-V)}^{2}-{({E}_{F}\cos \gamma \sin \phi +{A}_{y})}^{2}-{E}_{F}^{2}\,{\sin }^{2}\,\gamma \le 0$. In the simplest case *φ* = 0 and *γ* = 0, the tunneling is forbidden for the energy *E*_*F*_ < *V* + *A*_*y*_. Therefore, the transmission probability *T* = 0 when *E*_*F*_ < 56 meV, as shown the black solid line in Fig. 4(c). The incident angles *γ* and *φ* strongly affect the perfect transmission region, and therefore provide us with a possible way to control the transmission. We find that there are many transmission peaks at the low-energy regime induced by the electrostatic potential for antiparallel configuration (see Fig. 4(b,d)) since the low-energy transmission-forbidden region arises from the evanescent modes in the middle region 3. When the electrostatic potential *V*_3_ is large enough, the evanescent modes in region 3 are turned into the transmission modes. Such behavior offers us a possible way to construct an electric switching device.

### Transmission through double delta-function-shaped barriers

**Figure 5:**
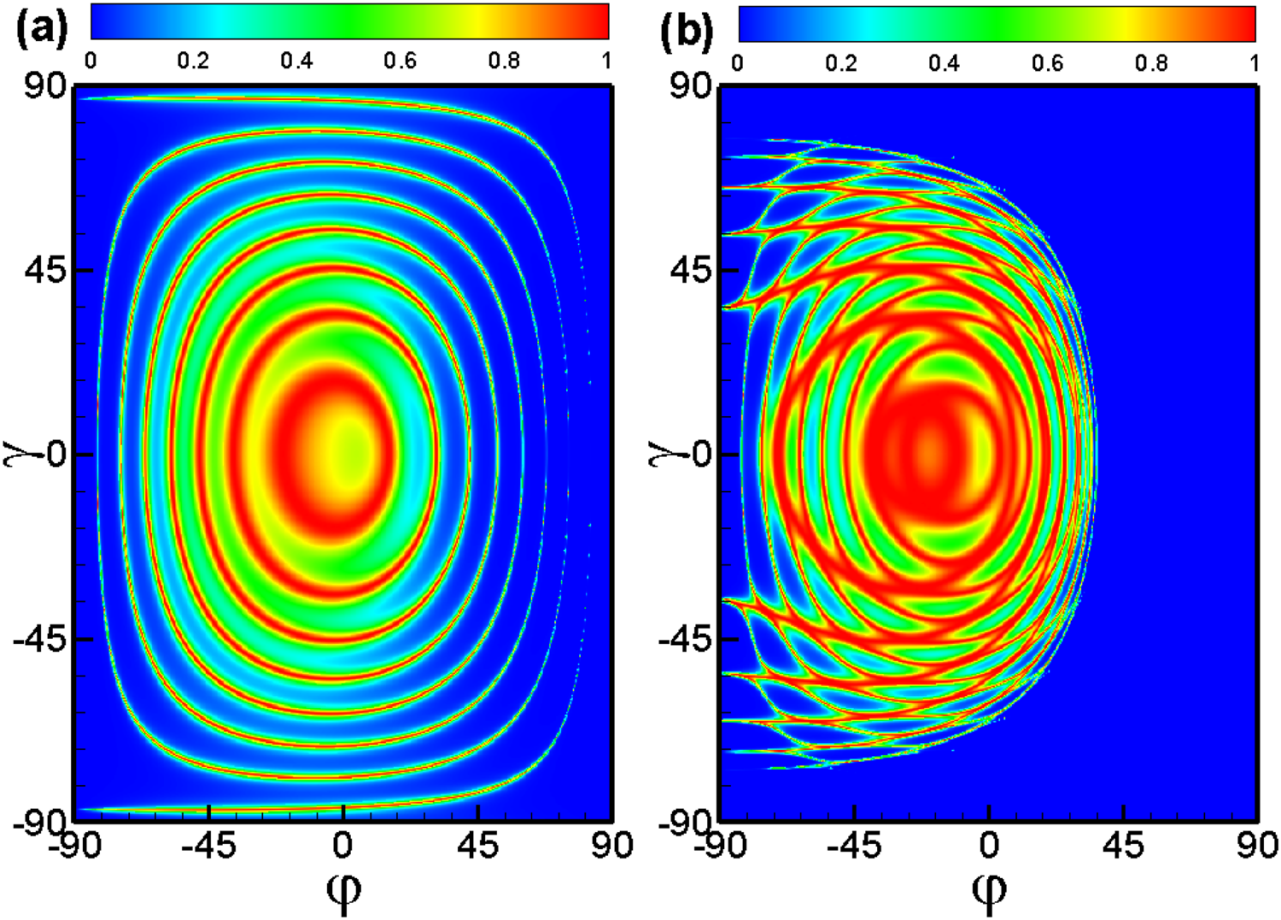
(**a**) The contour plot of the transmission probability through a parallel delta-function-shaped magnetic double barrier as a function of the incident angle *φ* and *γ*, incident energy *E*_*F*_ = 40 meV and barrier width *d* = 26 nm, distance *L* = 9*d*. (**b**) The same as panel (a) but for the barrier width *d* = 9*d*.

Now we consider tunneling through the delta-function-shaped magnetic barriers both for parallel and antiparallel configurations. For parallel configuration, the transmission spectra is symmetry about angle *γ*, as shown in Fig. 5. The Hamiltonian possesses a symmetry associated with the operation $\widehat{T}{\widehat{R}}_{z}{\widehat{\sigma }}_{y}$, where $\widehat{T}$ is the time-reversal operator, ${\widehat{R}}_{z}$ is the reflection operator about the *z* axis between the two barriers, and ${\widehat{\sigma }}_{y}$ is one of the Pauli matrices. This symmetry implies the invariance of the transmission with respect to the replacement *k*_*z*_ → −*k*_*z*_. The transmission exhibits obvious Fabry-Pérot resonant behavior for large positive incident angles which is very different from that for the square-shaped magnetic barrier case, where tunneling is totally forbidden for large positive incident angles see Fig. 2. As the barrier length *d* increases, the evanescent modes in the barriers reduce the transmission probability more significantly, and thus the resonant peaks in the region with large positive incident angles will disappear as shown in Fig. 5(b).

**Figure 6:**
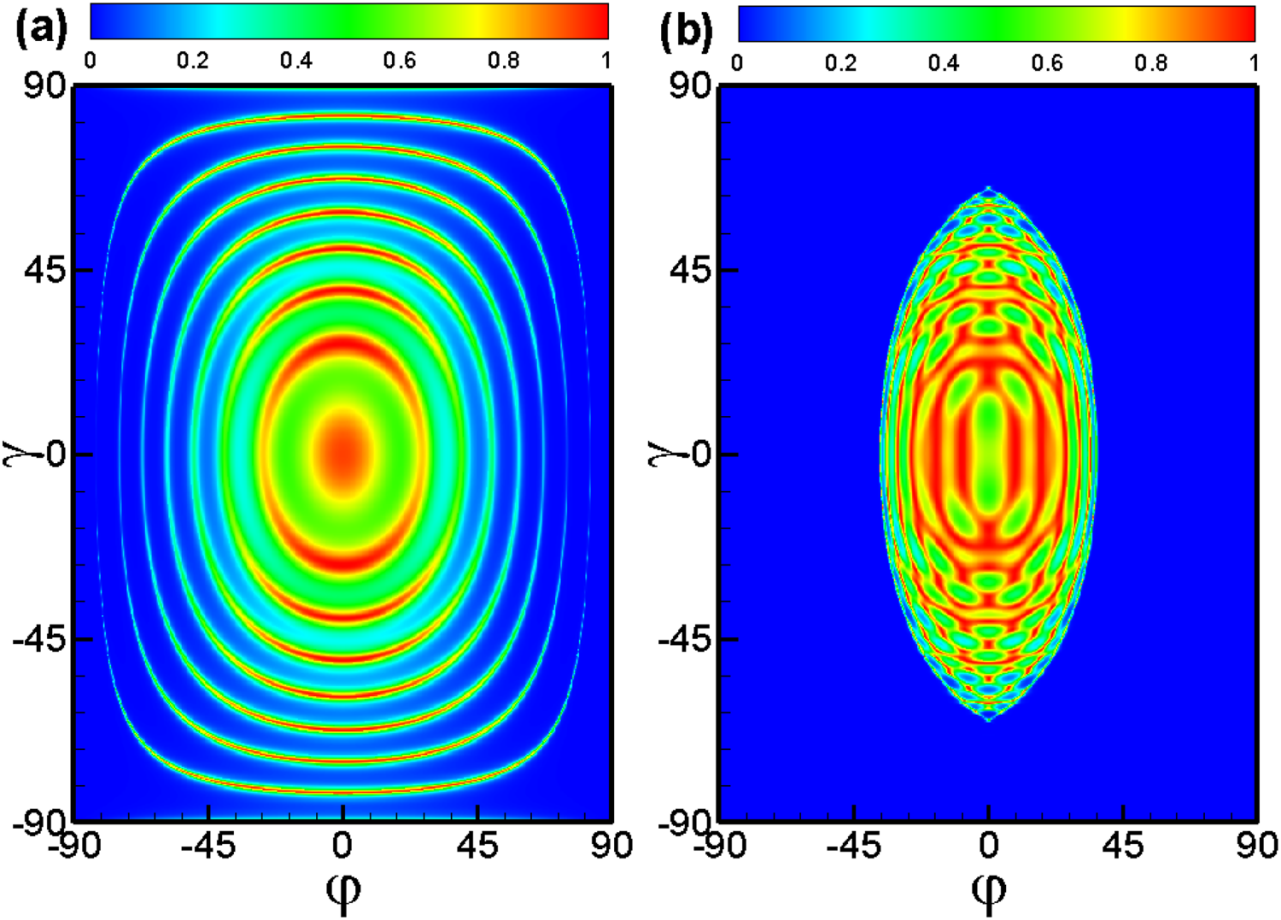
The same as Fig. 5 but for antiparallel configuration.

When we reverse the magnetization direction of the second ferromagnetic strip, the magnetization configuration is easily switched to the antiparallel configuration, as shown in Fig. 6. For this configuration, the vector potential is antisymmetric about the center of the whole structure. For antiparallel configuration, the transmission spectra is symmetry about angles *γ* and *φ*, which is different from that for parallel configuration. The Hamiltonian possesses symmetries associated with the operation $\widehat{T}\widehat{R}{\widehat{\sigma }}_{y}$ and $\widehat{T}{\widehat{R}}_{z}{\widehat{\sigma }}_{y}$, where $\widehat{R}$ is the reflection operator about the center between the two barriers. The two symmetries imply the invariance of the transmission with respect to the replacement *k*_*y*_ → −*k*_*y*_ and *k*_*z*_ → −*k*_*z*_. The parallel and antiparallel configurations strongly affect the perfect transmission region, and therefore provides us with a possible way to control the transmission by simply reversing the magnetization configuration of the ferromagnetic strips.

**Figure 7:**
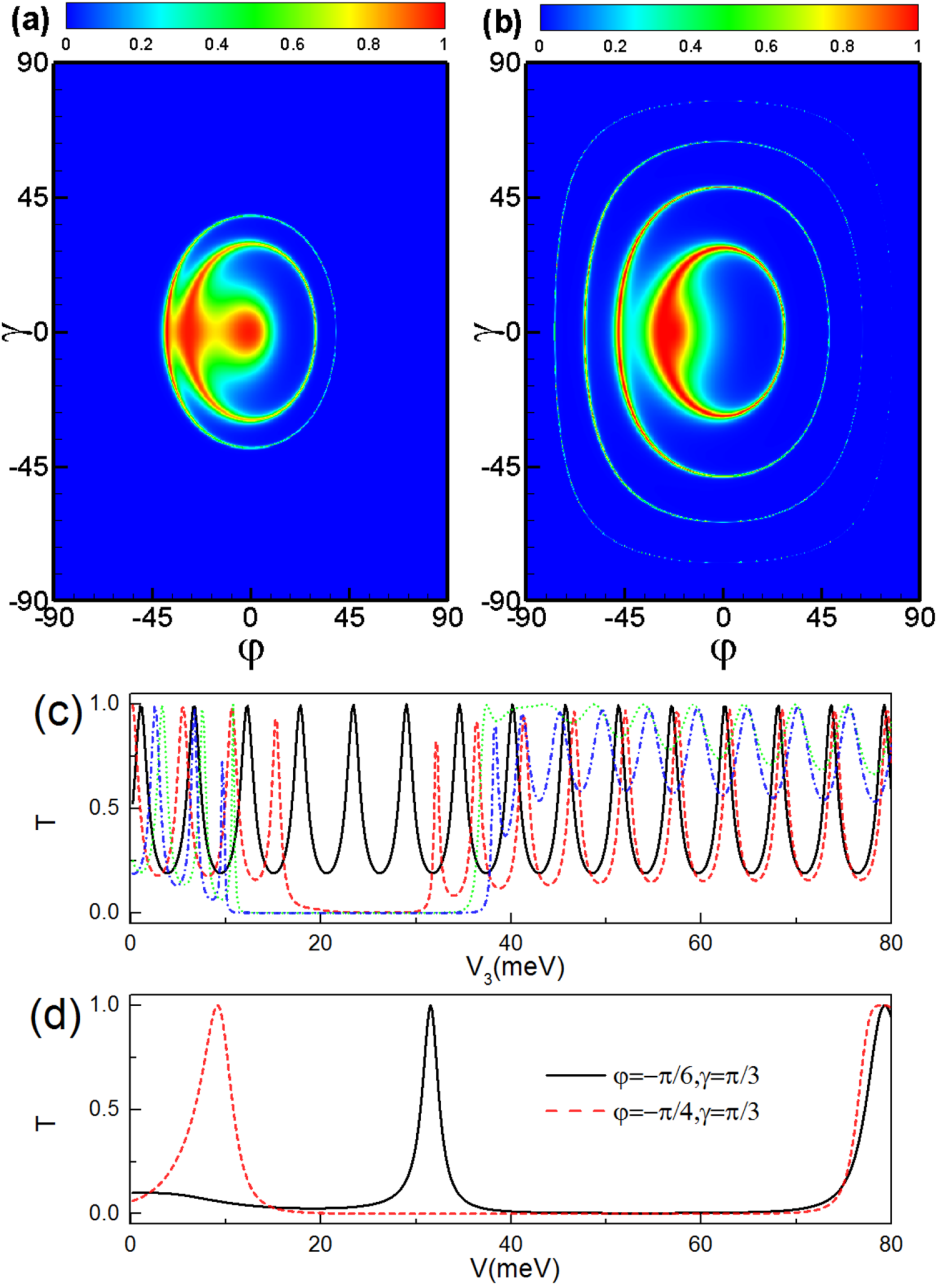
Transmission probability through a parallel delta-function-shaped magnetic double barrier as a function of the incident angle *φ* and *γ* with incident energy *E*_*F*_ = 24 meV and distance *L* = 9*d* = 234 nm for potential barrier *V*_3_ = 40 meV. (**b**) The same as panel (a) but for potential barrier *V*_2_ = *V*_4_ = *V* = 40 meV. (**c**) Transmission probability as a function of the potential barrier *V*_3_ for a different incident angle with incident energy *E*_*F*_ = 24 meV and distance *L* = 9*d* = 234 nm. The black solid line, red dashed line, green dotted, and blue dot-dashed line correspond to *φ* = 0 and *γ* = 0, *φ* = 0 and *γ* = *π*/12, *φ* = −*π*/6 and *γ* = 0, *φ* = −*π*/6 and *γ* = *π*/12, respectively. (**d**) The same as panel (c) but for the potential barrier *V*_2_ = *V*_4_ = *V*.

Next we discuss the transmission through a combined electric and delta-function-shaped magnetic double barrier. For parallel configuration, the transmission spectra is symmetry about angle *γ* in the presence of the potential barrier *V*_2_, *V*_3_, *V*_4_ (see Fig. 7). Interestingly, one can see that the transmission decreases even to zero as the incident angle increases. This is because evanescent modes appear when the incident energy approaches the height of the electric barrier, which suppresses the transmission in such incident angle interval. For delta-function-shaped magnetic double barrier with parallel configuration, *A*_*y*_ = 0 in the region 3 and 5, therefore the tunneling is forbidden satisfying ${\cos }^{2}\,\gamma \,{\sin }^{2}\,\phi +{\sin }^{2}\,\gamma \ge {({E}_{F}-V)}^{2}/{E}_{F}^{2}$. When apply the potential barrier in the region 3, i. e., *V* = *V*_3_, the transmission vanishes when the incident angle *φ* exceeds a critical value ${\phi }_{c}=\arccos \,({E}_{F}^{2}-{({E}_{F}-{V}_{3})}^{2})/({E}_{F}^{2}\,{\cos }^{2}\,\gamma )$ (see Fig. 7(a)) or ${V}_{3}\in ({E}_{F}-{E}_{F}\sqrt{{\cos }^{2}\,\gamma \,{\sin }^{2}\,\phi +{\sin }^{2}\,\gamma }$, ${E}_{F}+{E}_{F}\sqrt{{\cos }^{2}\,\gamma \,{\sin }^{2}\,\phi +{\sin }^{2}\,\gamma })$ (see Fig. 7(c)). Applying the potential barrier in the regions 2 and 4, i. e., *V* = *V*_2_ = *V*_4_, the total reflection for the parallel configuration stems from the evanescent modes in the middle region 2 and 4 and satisfies the relation ${({E}_{F}-V)}^{2}-{({E}_{F}\cos \gamma \sin \phi +{A}_{y})}^{2}-{E}_{F}\,{\sin }^{2}\,\gamma \le 0$. For a fixed incident energy, there is strong momentum-filtering behavior and can be tuned by the incident angles, as shown in Fig. 7(d).

**Figure 8:**
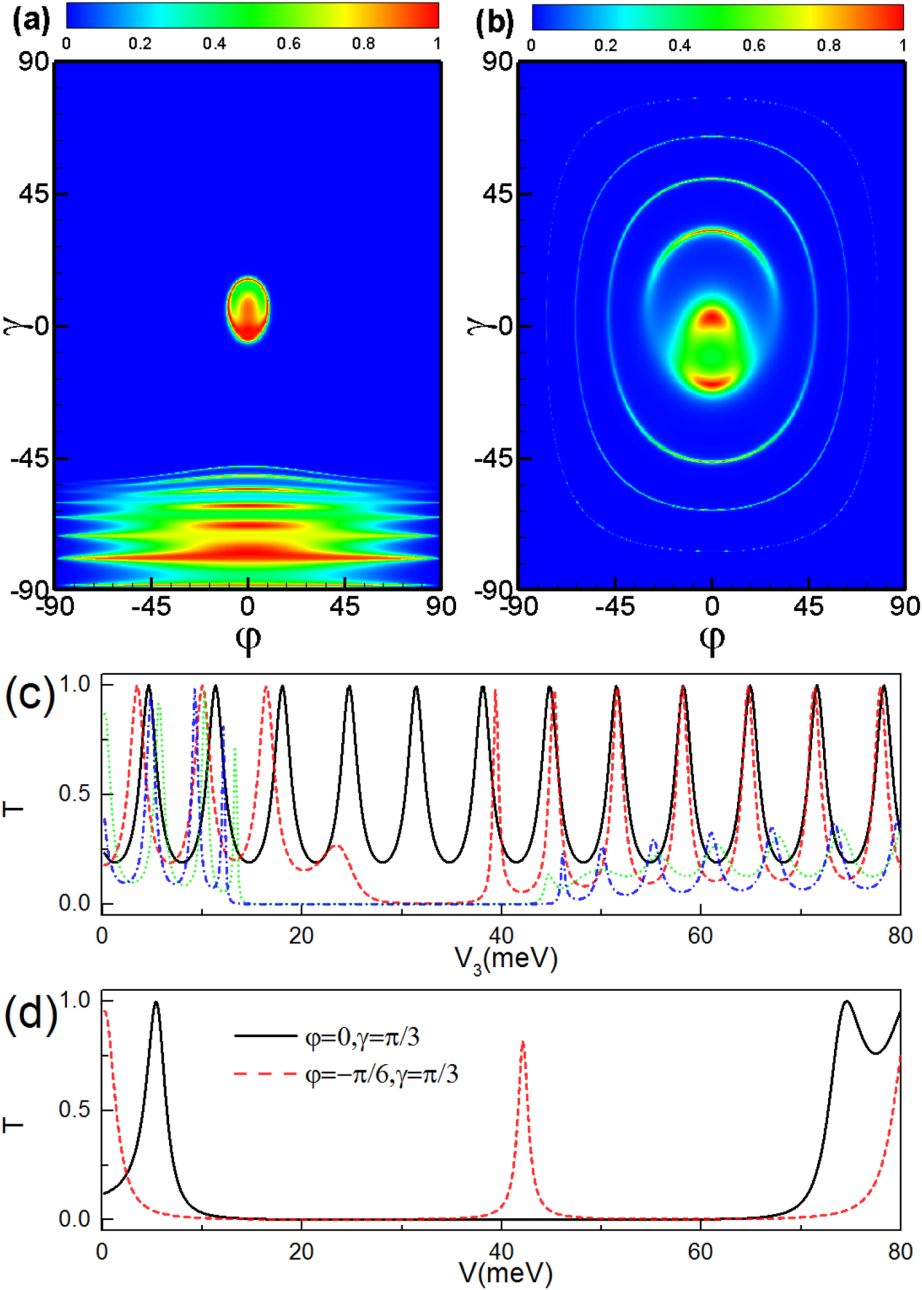
The same as Fig. 7 but for antiparallel configuration.

For antiparallel configuration, the transmission spectra is still symmetry about angle *φ* in the presence of the potential barrier *V*_2_, *V*_3_, *V*_4_ (see Fig. 8). We find that there are many transmission peaks at the regime *γ* ∈ (−90°, −45°) induced by the electrostatic potential (see Fig. 8(a)) since the transmission-forbidden region arises from the evanescent modes in the middle region 3. When the electrostatic potential *V*_3_ is large enough, the evanescent modes in region 3 are turned into the transmission modes. According to the relationship ${({E}_{F}-V)}^{2}-{({E}_{F}\cos \gamma \sin \phi +{A}_{y})}^{2}-{E}_{F}\,{\sin }^{2}\,\gamma \le 0$, the transmission peak can become more sharp for some incident angles (see Fig. 8(d)).

### The magnetoresistance

**Figure 9:**
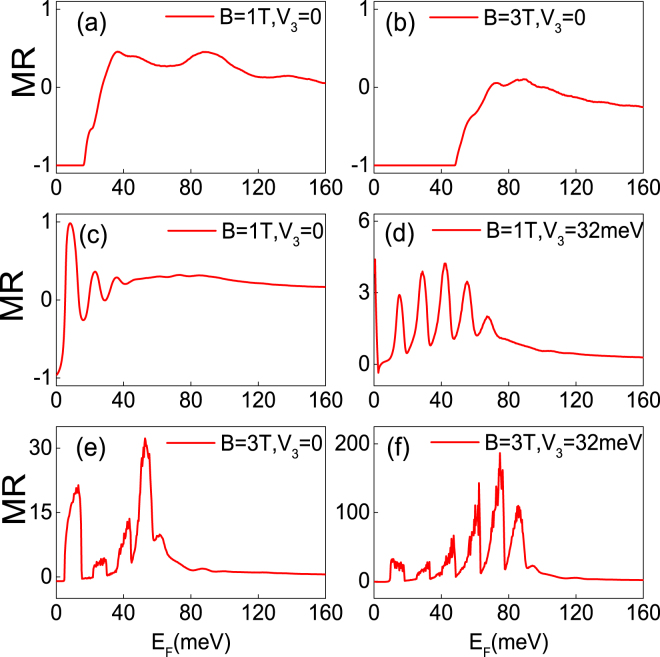
The magnetoresistance ratio MR as a function of the incident energy *E*_*F*_ in the presence of a double square-shaped magnetic/electric barrier, for *d* = 26 nm, *L* = 3*d*, *B* = 1 T, *V*_2_ = *V*_4_ = 0, *V*_3_ = 0. (**b**) The same as panel (a) but for *B* = 3 T. (**c**–**f**) The dependence on the incident energy of the MR in the presence of a double delta-function-shaped barrier, for *d* = 26 nm, *L* = 3*d*, (**c**) *B* = 1 T, *V*_3_ = 0; (**d**) *B* = 1 T, *V*_3_ = 32 meV; (**e**) *B* = 3 T, *V*_3_ = 0; (**f**) *B* = 3 T, *V*_3_ = 32 meV.

Finally, we focus on the magnetoresistance ratio *MR* = (*G*_*P*_ − *G*_*AP*_)/*G*_*AP*_, where the subscript *P* (*AP*) denotes parallel (antiparallel) configuration. The ballistic conductance is calculated from the Landauer-Bütiker formalism, $G={G}_{0}\,{\int }_{-\infty }^{\infty }\,{\int }_{-{k}_{F}}^{{k}_{F}}\,{\int }_{-{k}_{F}}^{{k}_{F}}\,TdEd{k}_{y}d{k}_{z}$, *G*_0_ = *e* ^2^ *L*_*y*_ *L*_*z*_/(*πh*) is taken as the conductance unit, *L*_*y*_, *L*_*z*_ are the sample size along the *y* and *z* direction which are much larger than *L* and *d*. In Fig. 9, the magnetoresistance ratio *MR* is plotted as a function of the Fermi energy for different heights of the electric and magnetic barriers. For a squareshaped double barrier, the magnetoresistance ratio *MR* is small over the calculated energy region as shown in Fig. 9(a,b). This feature is caused by the structure in the transmission discussed above. For a delta-function-shaped double barrier, the *MR*s exhibit significant oscillations and the peaks of *MR*s are corresponding to the peaks (valleys) of *G*_*P*_ (*G*_*AP*_) (see Fig. 9(c)–(f)). We also find that a strong magnetic field can effectively increase the magnetoresistance *MR*. Since a large magnetic field results in large imaginary wave vectors for the evanescent modes in the barrier region and strongly suppress the transmission probability. Therefore, the conductance *G*_*AP*_ at the valleys are close to zero and thus significantly increase *MR*. Note that the applied voltage *V*_3_ can also effectively increase the magnetoresistance *MR*. A giant magnetoresistance *MR* 200 can be achieved under the combined effects of the magnetic field and the applied voltage *V*_3_, as shown in Fig. 9(f). The electrostatic potential for the same magnetic barrier can shift the transmission gaps, enhance the difference of the transmission between parallel and antiparallel configurations, and thus can be used to adjust the *MR* efficiently.

## Conclusion

In summary, we study theoretically electron transport through planar magnetic barriers on the surface of a Weyl semimetal. We find that the electron transmission displays an interesting momentum-filtering feature, which can be controlled by tuning the incident angle, the Fermi energy, the magnetic field and the distance between the two barriers. The momentum filtering behavior becomes more significant for two-delta-function-shaped magnetic barriers. This behavior offers us an efficient way to control the transport and pave a way to construct Weyl semimetal-based electronic devices.
